# The Temporal Muscle of the Head Can Cause Artifacts in Optical Imaging Studies with Functional Near-Infrared Spectroscopy

**DOI:** 10.3389/fnhum.2017.00456

**Published:** 2017-09-15

**Authors:** Martin Schecklmann, Alexander Mann, Berthold Langguth, Ann-Christine Ehlis, Andreas J. Fallgatter, Florian B. Haeussinger

**Affiliations:** ^1^Department of Psychiatry and Psychotherapy, University of Regensburg Regensburg, Germany; ^2^Department of Psychiatry and Psychotherapy, Psychophysiology and Optical Imaging, University Hospital of Tübingen Tübingen, Germany

**Keywords:** fNIRS, NIRS, optical topography, noise, artifact, temporal muscle, clenching teeth

## Abstract

**Background:** Extracranial signals are the main source of noise in functional near-infrared spectroscopy (fNIRS) as light is penetrating the cortex but also skin and muscles of the head.

**Aim:** Here we performed three experiments to investigate the contamination of fNIRS measurements by temporal muscle activity.

**Material and methods:** For experiment 1, we provoked temporal muscle activity by instructing 31 healthy subjects to clench their teeth three times. We measured fNIRS signals over left temporal and frontal channels with an interoptode distance of 3 cm, in one short optode distance (SOD) channel (1 cm) and electromyography (EMG) over the edge of the temporal muscle. In experiment 2, we screened resting state fNIRS-fMRI (functional magnetic resonance imaging) data of one healthy subject for temporal muscle artifacts. In experiment 3, we screened a dataset of sound-evoked activity (*n* = 33) using bi-temporal probe-sets and systematically contrasted subjects presenting vs. not presenting artifacts and blocks/events contaminated or not contaminated with artifacts.

**Results:** In experiment 1, we could demonstrate a hemodynamic-response-like increase in oxygenated (O_2_Hb) and decrease in deoxygenated (HHb) hemoglobin with a large amplitude and large spatial extent highly exceeding normal cortical activity. Correlations between EMG, SOD, and fNIRS artifact activity showed only limited evidence for associations on a group level with rather clear associations in a sub-group of subjects. The fNIRS-fMRI experiment showed that during the temporal muscle artifact, fNIRS is completely saturated by muscle oxygenation. Experiment 3 showed hints for contamination of sound-evoked oxygenation by the temporal muscle artifact. This was of low relevance in analyzing the whole sample.

**Discussion:** Temporal muscle activity e.g., by clenching the teeth induces a large hemodynamic-like artifact in fNIRS measurements which should be avoided by specific subject instructions. Data should be screened for this artifact might be corrected by exclusion of contaminated blocks/events. The usefulness of established artifact correction methods should be evaluated in future studies.

**Conclusion:** Temporal muscle activity, e.g., by clenching the teeth is one major source of noise in fNIRS measurements.

## Introduction

Functional near-infrared spectroscopy (fNIRS) measures cortical activity by means of concentration changes of oxygenated (O2Hb) and deoxygenated (HHb) hemoglobin (Scholkmann et al., 2014). The measurement is spatially restricted to skull-near areas as the recording is done transcranially by fastening optic emitters and sensors (optodes) on the surface of the head. Near-infrared light that is detected at the sensor passed a banana-like shaped way (probability path) from the emitter to the sensor by penetrating skin, muscle, skull, cerebrospinal fluid, and brain tissue (Scholkmann et al., 2014). Changes in brain oxygenation due to cortical activity (neurovascular coupling) alter the absorption and scattering of the near-infrared light which is highly susceptible to O_2_Hb and HHb concentration changes (optical window) (Scholkmann et al., 2014). Neuronal activation is accompanied by locally specific increases in O_2_Hb and decreases in HHb. As the optodes are fixed to the head, the method is claimed to be relatively insensitive to movement artifacts, as they are induced for example in language tasks (Schecklmann et al., 2010). Furthermore, it is a “silent” recording technique (i.e., no scanner noise). These are advantages in contrast to functional magnetic resonance imaging (fMRI). However, whereas fMRI measurements are not confounded by oxygenation changes in the muscles or skin of the head, such extra-cerebral activity has been identified as a major source of noise in the fNIRS signal (Germon et al., 1998; Ferrari et al., 2004; Kirilina et al., 2012; Tachtsidis and Scholkmann, 2016). Cardiac, vascular, and respiratory activity affecting cortical and extra-cortical tissue is called global interference (Zhang et al., 2009). Proposed algorithms for artifact correction include frequency-specific filtering (Franceschini et al., 2003; Schroeter et al., 2004; Plichta et al., 2006, 2007a), subtraction of systemic noise derived from activity in areas of no interest (Franceschini et al., 2003; Haeussinger et al., 2014) or regression analyses of the pulse artifact (Gratton and Corballis, 1995). All of these correction algorithms are derived from the original fNIRS signal without using additional measurements. Shorter inter-optode distances are associated with a lower penetration depth of near-infrared light thus measuring rather the extra- than the intra-cerebral signal and enabling correction methods based on subtraction, regression, and adaptive filtering (Toronov et al., 2001; Zhang et al., 2007a,b, 2009; Luu and Chau, 2009; Virtanen et al., 2009; Gregg et al., 2010; Saager et al., 2011; Takahashi et al., 2011). Another bulk of studies used similar algorithms with regressors extrapolated from additional extra-cerebral measurements including laser Doppler flowmetry or pulse oximetry (Morren et al., 2004; Tachtsidis et al., 2008a,b, 2009; Takahashi et al., 2011; Kirilina et al., 2012, 2013).

Previous literature concentrated on the identification and correction of this non-specific global interference neglecting the influence of local oxygenation based on activity of head muscles. For example, in a recent review this issue is not discussed (Scholkmann et al., 2014). There is lot of literature investigating the influence and correction of head movements (Sato et al., 2006; Cui et al., 2010; Izzetoglu et al., 2010; Scholkmann et al., 2010; Cooper et al., 2012) which are accompanied by non-physiological trajectories (sharp onset, parallel change in O_2_Hb and HHb) due to relative movement between optodes and the head. However, head muscle activity itself results also in oxygenation changes in the muscle tissue. This issue is neglected in literature so far although there is evidence from sports medicine that fNIRS can be used to measure muscle oxygenation saturation (Ferrari et al., 2011). Recently, it could be shown that short muscle contractions provoke increases in O2Hb and decreases in HHb resembling cerebral oxygenation changes due to neural activity (Towse et al., 2011). Based on these observations and early evidence of multiple times increased blood flow in the jaw muscles after or during activation (Petersen and Christensen, 1973; Rasmussen et al., 1977), we suggest that the coupling of blood flow with activity in brain and muscle is governed by similar mechanisms.

One of the major head muscles is the temporal muscle covering the temporal bone with the main function of jaw movement. A large part of the fNIRS literature is based on verbal fluency tasks (Ehlis et al., 2014) and speech (Quaresima et al., 2012) which are accompanied by jaw movements. Thus, the question arises to what extent jaw movements contaminate fNIRS signals originating in the brain. One recent own study measured fronto-temporal fNIRS signals in a verbal fluency task (Schecklmann et al., 2010). We controlled for muscle activity by measuring electromyography (EMG) of the temporal muscle. Conditions were pronouncing and writing words with specific initial letters with paced answers. We did not find systematic associations of muscle activity as measured with EMG and fNIRS activity during verbal fluency. To our knowledge, this is currently the only study investigating the influence of temporal muscle activity on fNIRS signals. Here, we aimed to systematically investigate if temporal muscle activity as elicited by teeth clenching contaminates cortical fNIRS signals. Clenching teeth was identified as reliable and valid source of artifacts in preliminary measurements and own observations. This aim was realized by one experiment provoking a temporal muscle artifact and two experiments which were screened for temporal muscle artifacts offline.

In experiment 1, we instructed 31 healthy subjects to clench their teeth three times for 2 s. We measured oxygenation changes over the left fronto-temporal region by using a standard fNIRS probe-set and one short-optode channel to measure superficial oxygenation changes accompanied by recordings of temporal muscle activity. In experiment 2, we present a dataset of one subject who showed in a combined fNIRS-fMRI resting state measurement two temporal muscle artifacts. In experiment 3, we investigated sound-evoked auditory cortex activity in 33 healthy subjects in one block- and one event-related design.

## Material and methods

The study has been approved by the local ethics committee of University of Tübingen,(Germany; 252/2012BO1) and has been performed according to the Declaration of Helsinki. All participants gave written informed consent for study participation and publication of data without identifying information after a comprehensive explanation of the procedures. We conducted different experiments to show the influence of temporal muscle activity on fNIRS signal quality.

### Experiment 1: clenching teeth

First, we instructed 31 mentally and physically healthy subjects who reported no acute jaw and teeth problems (university students; age: 24.6 ± 2.8 years; sex: 14 females) to clench their teeth three times for 2 s. Moreover, subjects were repeatedly instructed to change breathing, to do physical exercise with hand exercise or to blink. Data of these randomized conditions are of no interest for the present study (breathing, physical exercise, and blink) and will be published elsewhere. The resting period after instruction to clench the teeth was 58 s. Instructions were presented via monitor in front of the subject.

**Figure 1:**
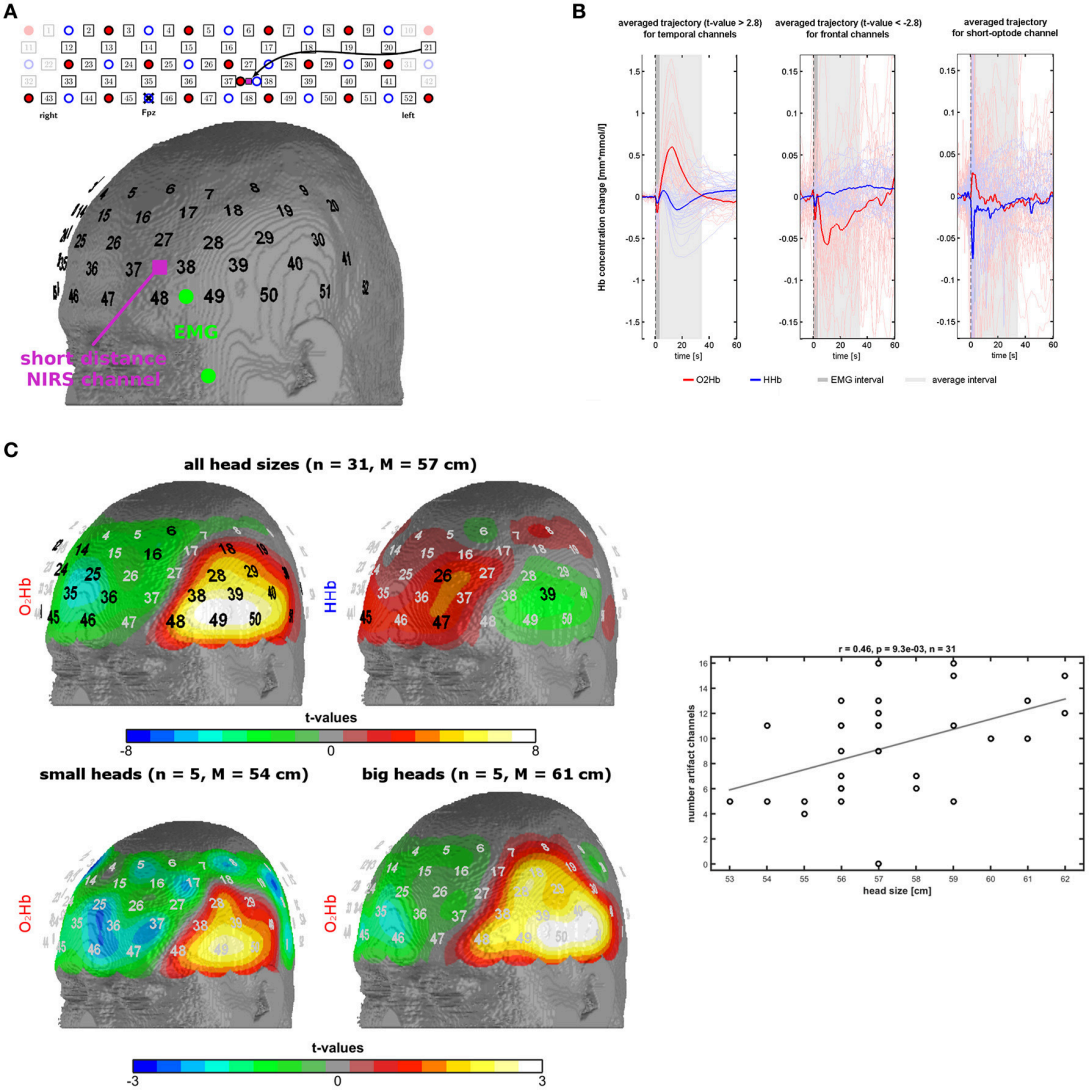
Measurement setup and findings of experiment 1. **(A)** Probe-set arrangement over the left frontotemporal area (red dots = light emitters, blue dots = detectors, numbers = channels), EMG electrodes in green, and short optode distance NIRS channel in pink. **(B)** Trajectories of O_2_Hb (red lines) and HHb (blue lines) in temporal and frontal channels of the probe-set showing significant changes. The dark gray box indicates a phase of an initial parallel dip of both chromophores induced by probe-set movement. The light gray box indicates the trajectory of the temporal muscle artifact which was used for further analyses. **(C)** Topographies of significant channels for O_2_Hb and HHb for all head sizes and for O_2_Hb for small and large head sizes. Scatterplot and correlation coefficient for the correlation between the number of artifact channels (spatial extent of the artifact) with head size. Please note that three dots are overlapping.

For the fNIRS measurement, we used a multi-channels continuous wave NIRS system (ETG-4000 Optical Topography System; Hitachi Medical Co., Japan) working with two different wavelengths (695 ± 20 and 830 ± 20 nm) and a time resolution of 10 Hz to measure relative changes of absorbed near-infrared light. These changes are transformed into concentration changes of O_2_Hb and HHb as indicators for brain activity by means of a modified Beer-Lambert law (Obrig and Villringer, 2003). The unit is mmol^*^mm/l, i.e., changes of chromophore concentration depend on the path length of the near-infrared light. We used one rectangular convoluted probe set (plastic panel) with three rows of light emitters and detectors (optodes). The arrangement of the probe set is shown in Figure 1. The probe set consisted of 15 light emitters and 14 detectors with an inter-optode distance of 3 cm. A measuring point of activation (channel) was defined as the region between one emitter and one detector. Thus, the probe set consisted of 44 channels and covered an area of about 24 × 6 cm on the scalp. The panels were fastened to the head by elastic straps. The probe sets were placed on the head with regard to the relevant standard positions of the international 10-20 system for EEG electrode placement (Jasper, 1958; Okamoto et al., 2004). The 4th optode in the bottom row of optodes was placed over Fpz with horizontal orientation along the line FPz-T3 with the probe set covering the left side of the forehead (three optodes on the right and seven optodes of the bottom optode line on the left hemisphere). We placed two additional optodes with 10 mm distance in between channel 37 and 38 resulting in a short distance (SD) channel using a self-made rubber mat. Brigadoi and Cooper suggest a distance of 8.4 mm in adults as optimal source-detector distance to measure extracortical signals (Brigadoi and Cooper, 2015). With 10 mm we chose the middle of discussed optimal short-optode distances (Goodwin et al., 2014). This additional channel is indicated to measure non-brain oxygenation changes over the edge of the temporal muscle. Placement of these SD optodes was done before the hairline as the signal was too noisy when optodes were placed over the belly of the temporal muscle which is covered by hair. Beside fNIRS channels we placed one ring electrode in front of the outer hair line over the edge of the temporal muscle and one ring electrode over the zygomatic bone to measure electromyographic activity (Palla and Ash, 1981) using a Brainamp ExG amplifier (Brain Products GmbH, Germany) (Figure 1A).

To investigate the trajectory of the artifact we applied a low-pass filter with a cut-off frequency of 0.5 Hz and calculated event-related averages for each subject and fNIRS channel using 10 s before the instruction as baseline. Based on visual inspection, we defined a time window of 3–34 s for further analyses (see Results). Shortly, the temporal muscle artifact shows a transient high-amplitude increase in O_2_Hb and parallel decrease of HHb with a latency in the range of tens of seconds. The average of the trajectory for this time window was calculated for each channel for further analyses. Channel-wise one-sample *t*-tests against zero were performed and the resulting *t*-values were mapped on the head surface of the Colin27 template (1993–2009 Louis Collins, McConnell Brain Imaging Centre, Montreal Neurological Institute, McGill University, Canada). Significant channels on a group level in these *t*-tests (*p* < 0.0021, Bonferroni-Holm corrected significance threshold) were used to identify the channels mainly affected by the artifact, referred to as artifact channels. For further analyses, we used the number of artifact channels or the mean signal of these artifact channels. In a next step, we analyzed the association of head circumference and spatial extent of the artifact by visually contrasting subgroups of subjects with small and big head sizes and correlating the number of artifact channels per subject with the head circumference. Rationale of this correlation analysis is that larger head sizes are associated with larger temporal muscles which again should result in larger areas of the temporal muscle artifact thus further validating that muscle activity is contaminating the fNIRS signal. The individual number of artifact channels was defined by visual inspection of the topography and the trajectory of the temporal muscle artifact (high amplitudes of O_2_Hb and HHb with anti-parallel run in temporal areas, for details see results). Note that artifact channels were defined on O_2_Hb and HHb concomitantly resulting in only one correlation of artifact and head size.

For analysis of the EMG signal, we filtered the raw EMG signal with a 0.05 Hz high pass to remove slow drifts. Thereafter, we calculated a moving standard deviation using a 100 sampling point window (which corresponds to 0.1 s). The moving standard deviation combines a down-sampling to 10 Hz (corresponding to a moving average), a mean correction for each bin (corresponding to a highpass-filter), and a rectification in one step. We calculated an event-related average for the three bite events and extracted the peak, the latency of the peak, and the mean value of the averaged EMG response for the time window 0–3 s for each subject as measures for the muscle activity. We analyzed the association of the EMG and the fNIRS artifact (mean signal over all artifact channels) by correlating the peak, the latency of the peak, and the mean amplitude of both signals respectively. Peak of the fNIRS artifact was obtained by conducting a peak detection for the time window between 3 and 34 s after the biting event.

For analysis of the hemodynamics in extra-cranial layers we analyzed the SOD channel in analogy to the LOD channels using a 10 s baseline and calculating the average signal from 3 to 34 s after the instruction. We correlated for each single subject trajectories of the fNIRS muscle artifact and the SOD signal for the time window 3–34 s. On a group level, we correlated the peak, the latency of the peak, and the mean amplitude of the SOD activation and the fNIRS muscle artifact, respectively. Correlation of EMG and SOD signal with the fRNIS muscle activity was repeated with head size as covariate to see if head size has influence on the correlations.

### Experiment 2: fMRI of temporal muscle

Here, we present a dataset of one subject who showed in a combined fNIRS-fMRI resting state measurement two temporal muscle artifacts, i.e., large transient O_2_Hb increases and HHb decreases with a duration longer than 30 s. Magnetic resonance imaging was conducted using a 3 T Siemens MAGNETOM Trio scanner. A structural image with a resolution of 240 × 256 × 159 voxels and a voxel size of 1.0 × 1.0 × 1.0 mm^3^ was obtained by applying a T1 weighted MP-RAGE sequence (3D magnetization prepared rapid gradient echo, TR = 2,300 ms, TE = 2.92 ms) for each subject. During a resting state task, functional images with a resolution of 80 × 80 × 29 voxels and a voxel size of 2.5 × 2.5 × 3.5 mm^3^ were acquired applying a multi-echo planar imaging (mEPI) sequence (TR = 2,500, TE1 = 15 ms, TE2 = 37 ms, flip angle = 90°). According to Richard and colleagues the T2 relaxation time of the skin layer is smaller 30 ms (Richard et al., 1991). For gray matter in the human frontal cortex Wanspura and colleagues reported a T2 value of 41 ms (Wansapura et al., 1999). Since an optimal BOLD-sensitivity is achieved for maximal tissue MR-intensity (Deichmann et al., 2002), we chose the TE1 EPI time series to analyse the BOLD-contrast in skin voxels and the TE2 to analyse the BOLD-contrast in the brain. The first four volumes were discarded to account for magnetization saturation effects.

Simultaneously, fNIRS was conducted using eight emitters and eight detectors attached in a quadratic arrangement on the right frontal head of the participant also covering partly (three optodes) the temporal region. The lateral optode of the bottom row was placed over Fpz with direction of this row toward T4. Other technical specifications are identical to experiment 1 of the present work.

The structural image was segmented into air, scalp, skull, cerebral spinal fluid (CSF) as well as gray and white brain matter using SPM8 toolbox (http://www.fil.ion.ucl.ac.uk/spm/). To extract the temporal muscle from the structural scan we first masked the scan with the scalp mask resulting from the anatomic segmentation. In a transversal slice view the fat-rich hypodermis, i.e., the deepest scalp layer, could be clearly seen as a ring of high intensity values surrounded by the remaining superficial scalp layers. In the temple region, there were clusters of scalp-segmented low intensity voxels that were attached *inside* the hypodermis ring. The anatomic location and structure suggested that these voxels represent the temporal muscle. The voxels were automatically selected using a combined intensity and location (deeper than the bright hypodermis ring) criterion.

**Figure 2:**
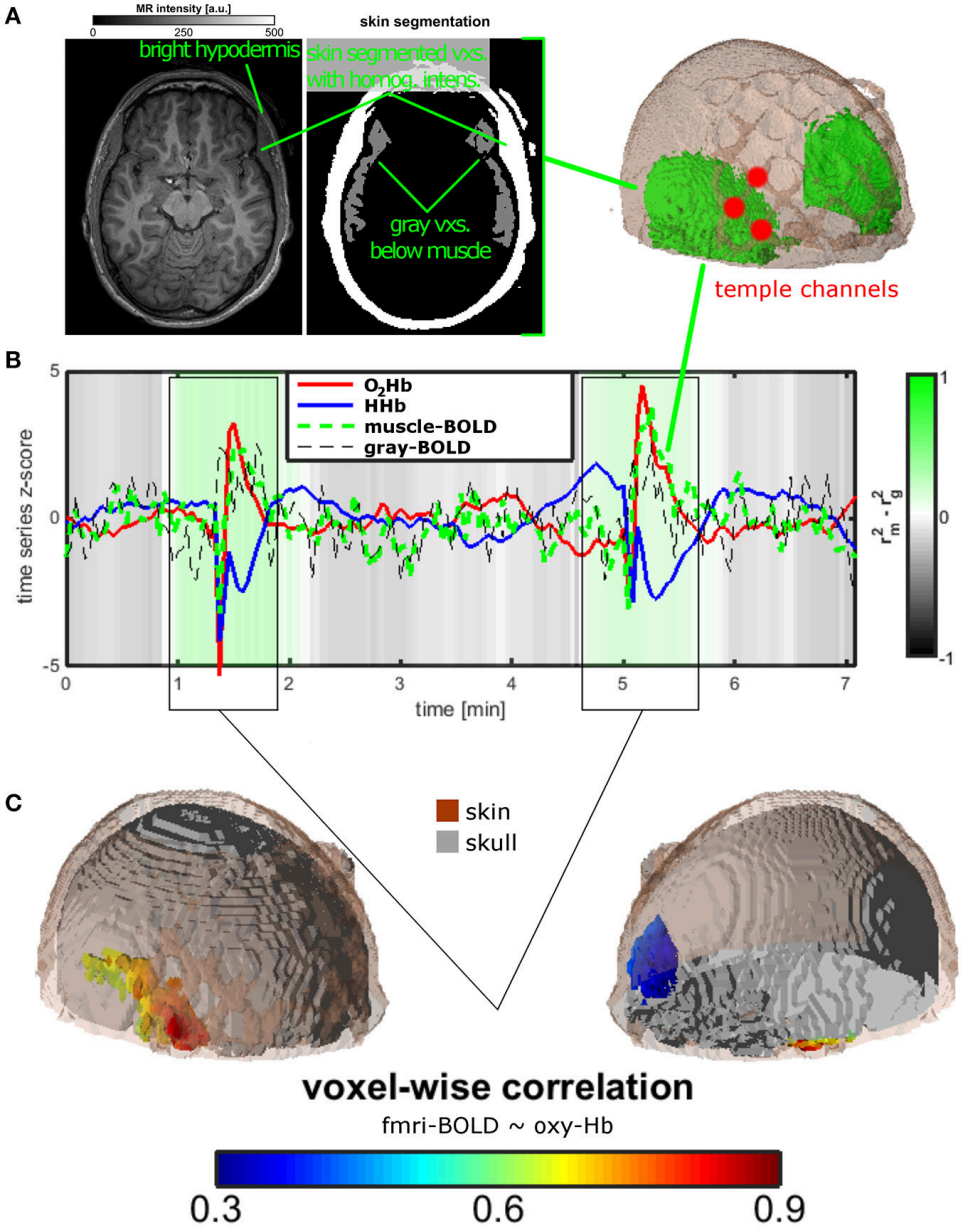
Measurement setup and findings for experiment 2. **(A)** Temporal muscle extraction of one healthy subject and three fNIRS channels covering the edge of the temporal muscle. **(B)** Resting state measurement of the healthy subject. Mean trajectories of O_2_Hb and HHb of the three fNIRS channels, of muscle and gray matter BOLD of extra- and intracranial voxels in the area of the temporal muscle. The gray-green color bar indicates the relationship of the explained variance of the correlation between O_2_Hb and muscle and between O_2_Hb and gray matter BOLD signal. Based on visual inspection and correlation analyses, two time windows were identified showing the temporal muscle artifact. **(C)** For the two time windows of the artifact, O_2_Hb signal was correlated with all voxels showing correlations in extra-cranial layers mirroring the temporal muscle on both head sides (lower on the right side, indicated by blue color).

We transferred the muscle voxels to the EPI-space using the affine transformation matrix (thus, distortions and signal losses of the EPI scans were omitted) and extracted a muscle-BOLD time series for all voxels of the temporal muscle (Figure 2A). Analogously to this procedure we extracted a gray-matter-BOLD time series from voxels that were located in the cortex beneath the muscle (Figure 2A). For the fNIRS signal, we extracted the average time course for O_2_Hb and HHb for the three channels covering the temporal muscle. Each time series, i.e., EPI-BOLD as well as O_2_Hb and HHb, were bandpass filtered with a pass window of 1/120–1/10 Hz. Figure 2B shows time courses for O_2_Hb, HHb, muscle-, and gray-matter-BOLD. To investigate the temporal association between fNIRS and fMRI signals, we correlated the O_2_Hb time courses with the time course of gray-matter- and muscle-BOLD using a sliding correlation with a window of 60 s. Gray-green coloring shows the difference in explained variance between the correlation of O_2_Hb and muscle and the correlation of O_2_Hb and gray matter. Based on these correlations and visual inspection of the time courses of O_2_Hb and HHb we were able to define two time windows with clear temporal muscle artifacts. To investigate the spatial association of O_2_Hb and fMRI signals, we correlated the O_2_Hb signals with each EPI-voxel independent from the extracted layer for the two defined time windows contaminated with the muscle artifact (Figure 2C). Analyses for HHb showed similar findings as for O_2_Hb and were not reported.

### Experiment 3: sound-evoked fNIRS of temporal cortex

We investigated sound-evoked auditory cortex activity in 33 healthy subjects (university students; age: 32.1 ± 12.7 years; sex: 15 females) as we had clear hypotheses about the spatial localization of sound-induced activation (Plichta et al., 2011; Schecklmann et al., 2014). Furthermore, this area is partly covered by the temporal muscle. Parts of this sample and the design of this study were already published (Schecklmann et al., 2014). We used one block- and one event-related design for measurement of oxygenation changes induced by acoustic stimulation. “Comité Consultatif International Télégraphique et Téléphonique” (CCITT) speech noise was presented binaurally by means of insert earphones (E-A-RTONE3A, Aero Company, USA). The tip of the earphones was placed into the auditory canal, guaranteeing an exact adjustment of the sound intensity. Intensity level was set to 70 dB SPL. For the block design, participants listened to 12 blocks of CCITT noise. Each block lasted 20 s and was followed by a 20 s resting period. For the event-related design, stimuli were presented 40 times with a jittered inter-stimulus interval of 12–14 s for 1.75 s.

**Figure 3:**
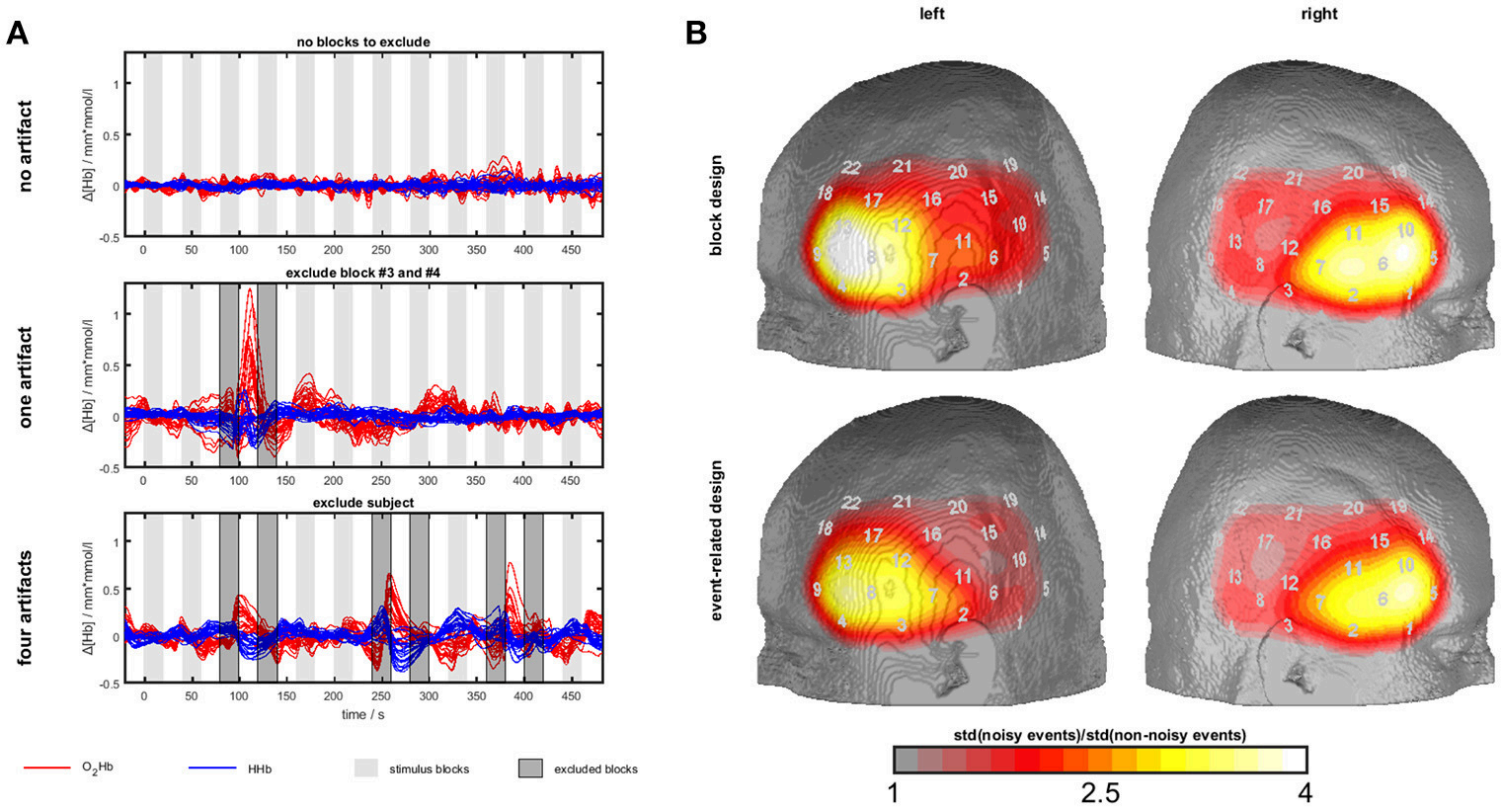
Temporal muscle artifacts for experiment 3. **(A)** Exemplary fNIRS data with no, one and four artifacts resulting in the exclusion of none, two and seven blocks, respectively, from data analysis. **(B)** Contrasts of the standard deviation of events/blocks with and the standard deviation of events/blocks without artifacts in a channel-wise manner in the group of subjects with artifacts showing the typical topography of the temporal muscle artifact.

We used two identical rectangular probe sets with eight light emitters and seven detectors for each NIRS probe set. Thus, one probe set consisted of 22 channels and covered an area of 6 × 12 cm on the scalp. The channel over the middle lower optode was placed over T3/T4 with vertical orientation in direction to C3/C4. As functional region of interest (ROI) for the auditory stimulation, we defined the auditory cortex (Brodmann area; Kirilina et al., 2013; Goodwin et al., 2014; Brigadoi and Cooper, 2015) and Broca's area (Brodmann are Richard et al., 1991; Wansapura et al., 1999). See Figure 3 for the probe set arrangement and the channel-wise coverage probability of the ROIs (probability mapping was done in accordance to the methods of Singh et al., 2005). Channels with a probability greater than 0.5 were marked. Each ROI value was obtained by calculating the probability-weighted sum of all channel values. For the auditory ROI 9 channels per hemisphere had a probability greater than 0. For the Broca's area there were 7 channels on the right and 6 channels on the left hemisphere with a probability greater 0.

To detect muscle artifacts, we conducted a visual inspection of the O_2_Hb and HHb time series of all channels for each subject. Based on data from experiment 1, a muscle artifact as elicited by clenching the teeth can be easily identified by its large amplitude and its characteristic long-latency shape with O_2_Hb and HHb comprising an anti-parallel run. Additionally, a strong initial artifact (sudden parallel signal change of O_2_Hb and HHb) is often visible. Figure 3A shows typical examples. This way, we divided the sample into two groups, i.e., subjects with and without muscle artifacts. Subjects with more than 50% contaminated blocks/events were excluded from further analyses (*n* = 5 for block and *n* = 3 for event-related design). To derive topographical information of the artifact trajectory, we contrasted the standard deviation of events/blocks with and the standard deviation of events/blocks without artifacts in a channel-wise manner in the group of subjects with artifacts. **Figure 5** shows the ratio of noisy to un-noisy blocks/events.

Neural activation was inferred by means of a model-based approach. We used the SPM8 hemodynamic response function (HRF) with a peak time of 6 s. As we found two different types of hemodynamic responses for the block design we conducted two independent regression analyses: one with a simple HRF and one with a HRF convolved with a 15 s boxcar function for each block. For the event-related design we used the simple HRF. Beta-weights were estimated by means of least-square linear regression considering a first-order auto-regression model. To obtain a measure for neural activation, one-sample *t*-tests against zero were calculated for each fNIRS channel across subjects. The alpha-level of 0.05 was adjusted by the Bonferroni-Holm procedure to account for multiple testing.

To show relevance of temporal muscle artifacts, we contrasted activation of groups with and without artifacts and conditions with and without artifacts excluding the noisy blocks/events (Student *t*-tests for independent and dependent samples). Activation was defined as the *t*-value against zero.

## Results

### Experiment 1

#### Topography and trajectory of the temporal muscle artifact

Based on visual inspection, we could characterize the temporal muscle artifact showing significant channels over the temple area with a trajectory of increases of O_2_Hb and decreases of HHb from 3 s after the instruction to clench the teeth and returning to baseline level at 34 s. Trajectories are shown in Figure 1B, and t-maps are shown in Figure 1C. For the time window 3-34 s, O_2_Hb showed an increase followed by a decrease to baseline level. HHb showed a decrease followed by an increase to baseline level with an initial overshoot. The beginning (3 s) and the end of the analysis window (34 s) were defined by the zero-crossings of O2Hb and HHb both crossing each other at these time points. Initially, O2Hb and HHb showed parallel negative dips for about 3 s.

Figure 1C shows the t-maps for the changes in O_2_Hb and HHb induced by the temporal muscle artifact as indicated by the average signal for the time window 3-34 s. Two main regions can be identified: (1) The temple region with significant positive and negative *t*-values for O_2_Hb and HHb, respectively, and (2) the frontal region with significant negative and positive *t*-values for O_2_Hb and HHb, respectively. Notice the y-scale, which is ten times larger for the temporal than for the frontal region. Notice also the parallel initial dip of O_2_Hb and HHb in the frontal area. The frontal region shows a reversed signal pattern with less smooth and longer trajectories, especially for O_2_Hb.

#### Head size and topography of the artifact

In the bottom part of Figure 1C, it can be recognized that there is a difference between head sizes (five subjects per group with head circumference <56 cm and >59 cm respectively) regarding the spatial extent of the artifact. Larger head circumference is associated with larger topographies. On a metric scale (number of artifact channels per subject) head size and artifact size is positively correlated (*r* = 0.46; *n* = 31; *p* = 0.009) as shown in Figure 1C (bottom).

#### EMG muscle activity and fNIRS artifact

Table 1 shows the correlations of the average, peak and latency of peak activity between the EMG and the fNIRS signals indicating no significant association except the peak of EMG activity and peak of O_2_Hb activity.

**Table 1:** Correlation of measures of EMG and SOD activity with the corresponding measures of fNIRS activity in the artifact area (without and with head size as covariate).

**EMG**	**O_2_Hb**	**HHb**
Average signal	*r* = 0.03; *p* = 0.89	*r* = 0.24; *p* = 0.20
Peak of signal	*r* = 0.41; *p* < 0.05	*r* = –0.24; *p* = 0.19
Latency of peak of signal	*r* = –0.19; *p* = 0.30	*r* = 0.03; *p* = 0.88
**SOD**	**O**_2_**Hb**	**HHb**
Average signal	*r* = 0.21; *p* = 0.27	*r* = –0.14; *p* = 0.45
Peak of signal	*r* = 0.02; *p* = 0.92	*r* = –0.17; *p* = 0.35
Latency of peak of signal	*r* = 0.43; *p* < 0.05	*r* = –0.41; *p* < 0.05
**WITH HEAD SIZE AS COVARIATE**		
**EMG**	**O**_2_**Hb**	**HHb**
Average signal	*r* = 0.02; *p* = 0.90	*r* = 0.24; *p* = 0.20
peak Of signal	*r* = 0.41; *p* < 0.05	*r* = –0.24; *p* = 0.19
Latency of peak of signal	*r* = –0.18; *p* = 0.35	*r* = 0.02; *p* = 0.91
**SOD**	**O**_2_**Hb**	**HHb**
Average signal	*r* = 0.17; *p* = 0.36	*r* = –0.14; *p* = 0.46
Peak of signal	*r* = –0.04; *p* = 0.83	*r* = –0.18; *p* = 0.35
Latency of peak of signal	*r* = 0.44; *p* < 0.05	*r* = –0.41; *p* < 0.05

#### Extracranial signals and fNIRS artifact

On a single subject level, we correlated the time course of the muscle artifact with the time course of the SOD channel for the time window of 3–34 s. Correlation coefficients showed high variability with most of the subjects showing positive correlation coefficients (*n* = 28). High effects sizes defined by correlations coefficients over 0.5 were only found in 19 subjects for O_2_Hb and ten subjects for HHb. On a group level, different measures (amplitude, peak, latency of peak) of SOD and muscle artifact activity were not correlated except the correlation of peak of EMG and peak of O_2_Hb signal and the latency of the SOD and the latency of the O2Hb and HHb signal (Table 1). Including head size as covariate did not change this findings.

### Experiment 2

Figure 2 summarizes the results for the single-case fNIRS-(f)MRI-experiment. Figure 2B shows the mean time course of O_2_Hb and HHb of three fNIRS channels covering the temporal muscle and of the BOLD-signal in muscle tissue of and gray matter below the temporal muscle and as extracted by structural MRI (Figure 2A). Statistics in this figure are shown only for O_2_Hb as HHb showed the same pattern. Gray-green coloring shows the difference in explained variance between the correlation of O_2_Hb and muscle and the correlation of O_2_Hb and gray matter. Over the whole measurement, correlation of O_2_Hb and gray matter is higher than the correlation of O_2_Hb with muscle tissue indicating valid measurement of brain oxygenation with fNIRS. For two time windows (see also below), this ratio is reversed indicating the measurement of muscle oxygenation with fNIRS. Time courses of O_2_Hb and HHb showed high-amplitude anti-parallel changes of both chromophores showing the typical muscle artifact as described in experiment 1. Based on the correlation analyses and visual inspection, we defined two artifact time windows (0:55–1:54 and 4:38–5:40 min). For these time segments, voxel-wise correlation between the O_2_Hb and EPI-voxel BOLD signals revealed high correlation coefficients in voxels located in the extra-cranial temple-region of the right hemisphere (Figure 2C). Considering the muscle extraction (Figure 2A), these voxels could be clearly assigned to the temporal muscle.

### Experiment 3

Firstly, we identified sub-groups with (block design: *n* = 14; event-related design: *n* = 18) and without (block design: *n* = 14; event-related design: *n* = 12) visually detectable artifacts. Several subjects were excluded due to more than 50% of blocks/events with muscle artifacts (*n* = 5 for block and *n* = 3 for event-related design). Within the group of subjects with artifacts, we plotted the channel-wise ratio of the standard deviation of blocks/events with and without artifacts in Figure 3B. The standard deviation is up to four times higher for the blocks/events with artifacts. The most effected region covers inferior frontal parts of the probe set which is corresponding to the area of the temporal muscle (compare experiment 1 and 2) validating the present visual artifact detection.

**Figure 4:**
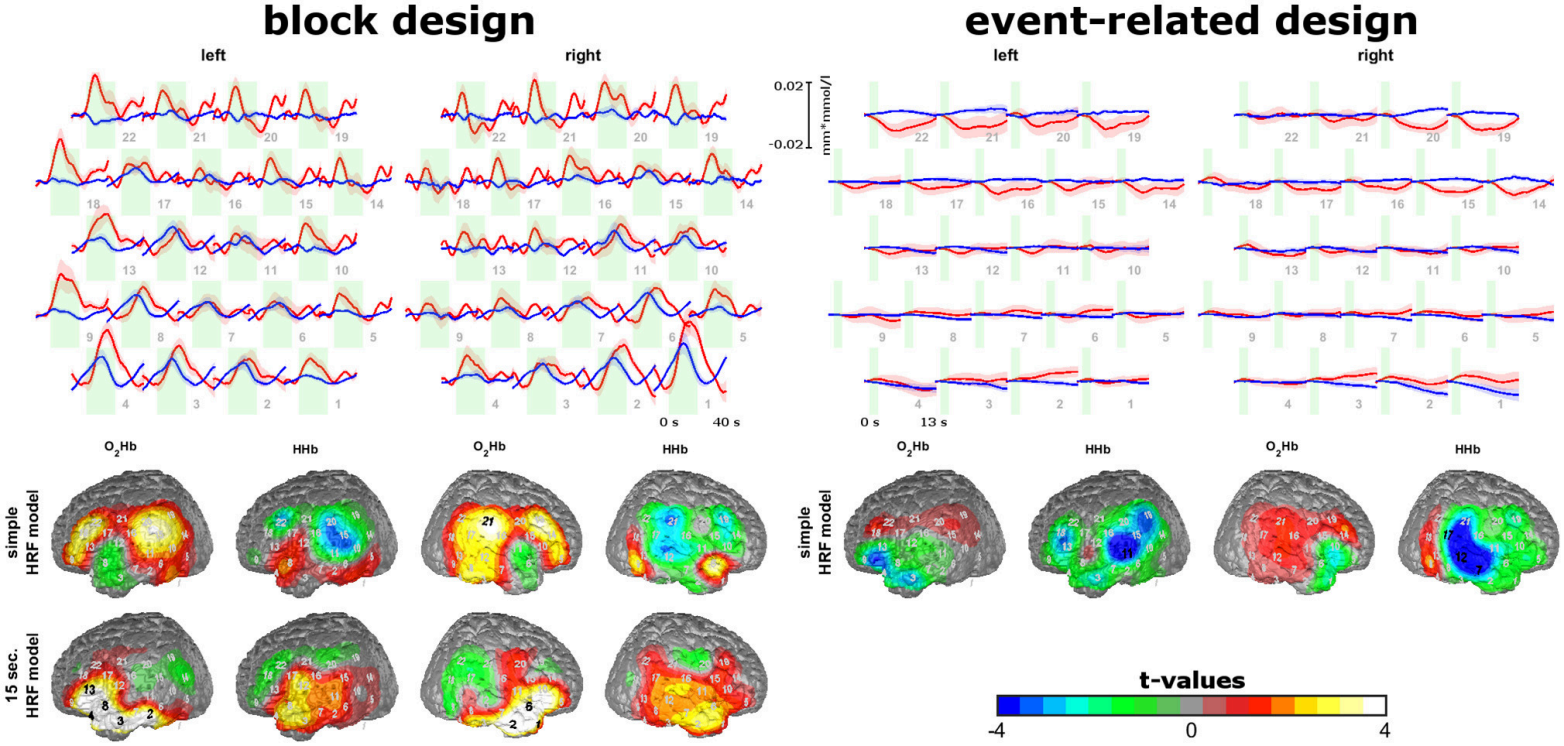
Sound-evoked activity of experiment 3. Trajectories of O_2_Hb (red lines) and HHb (blue lines) for the block and event-related design are shown on the top. Topographies of model-based analyses of block and event-related design are shown on the bottom. Please note, that for block design two model-based approaches were analyzed. Findings show short-lasting increases in areas over the auditory cortex and Broca's area for the block and event-related design and stable increases over the 20 s of auditory stimulation in inferior frontal areas for the block design.

For the group of subjects without artifacts, trajectories and topographies for the block and event-related design are shown in the top of Figure 4. As oxygenation showed different peak times and latencies in different channels for the block design, we analyzed data with a simple HRV model for the block and event-related design but also using a box-car function with 15 s for the block design. For the block design and the event-related design using the simple HRF model, hemodynamic responses (increases in O_2_HB and decreases in HHb) are visible in channels covering the auditory ROI and Broca's area. For the block design, activation was transient within the first 10 s of sound stimulation which lasted 20 s. For the event-related design, small initial activation can be detected after the short sound stimulation. Here, activation of the auditory ROI seems to be valid only for HHb and not for O_2_Hb as HHb is more focal in contrast to O_2_Hb (right bottom of Figure 4). For the block design, using the box-car function, inferior anterior channels of the probe set showed increases of O_2_Hb and HHb with a stable plateau of activation over the whole course of the 20 s sound stimulation.

**Figure 5:**
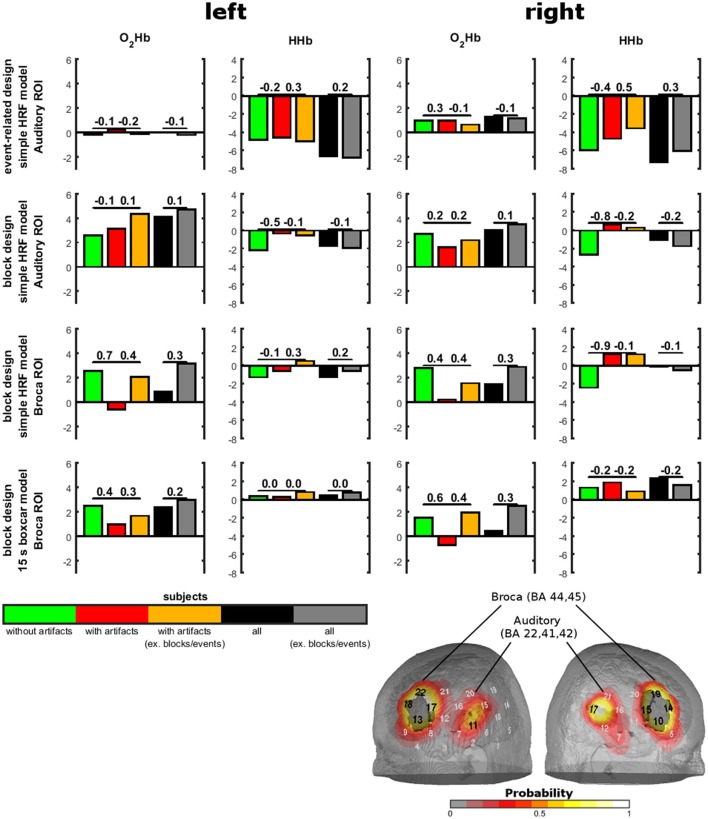
Sound-evoked activity in anatomically defined regions of interests (ROIs) of experiment 3. Anatomically defined ROIs were the auditory cortex and Broca's area as shown on the right bottom. Sound-evoked activity (bars) in these areas was defined by *t*-tests against 0. Contrasts between groups with and without artifacts and between conditions with and without exclusion of artifacts are shown by effect sizes over the bars.

To show the relevance of temporal muscle artifacts, we contrasted auditory cortex and Broca's area activity of groups with and without artifacts and within the group showing artifacts conditions with and without artifacts excluding the noisy blocks/events (Figure 5). No contrast was significant (all *p*-values > 0.1). Thus, to estimate statistical power we provide effect sizes for the contrasts. For the event-related design, O_2_Hb showed no valid activation. For HHb, excluding artifacts (by excluding noisy events or contrasting groups with and without artifacts) showed small effect sizes for the left auditory ROI and medium effect sizes for the right auditory ROI. Subjects without artifacts showed the highest activation. Interestingly, the group of subjects without artifacts did not show an increase in strength of activation. For the block design and the simple HRV model, subjects without artifacts showed O_2_HB increases and HHb decreases in the auditory ROI. For O_2_Hb, artifact correction only resulted in small effect sizes while for HHb, activation was reduced with medium to high effect sizes for the group of subjects showing artifacts independent from excluding noisy blocks. For Broca's area using the simple HRV model, activation can be detected in the group of subjects without artifacts for O_2_Hb (left and right hemisphere) and HHb (right side only). For HHb of right Broca's area, the group of subjects with artifacts showed diminished activation which could not be recovered by excluding blocks with artifacts. For O_2_Hb, activation was diminished for participants with artifacts (medium to high effect sizes) which was partly recovered by excluding noisy blocks (medium effect sizes). For the block design using the boxcar model, activation was visible only for O_2_Hb which was diminished for the subjects with artifacts and recovered by excluding noisy blocks.

Inspecting effect sizes for the group including all subjects (*n* = 28 for block design, *n* = 30 for event-related design), differences between the conditions including artifacts and excluding artifacts showed only small effects. Otherwise, in specific analyses excluding artifacts turned non-significant *t*-tests significant by increasing the *t*-value over the significance threshold which is at about T = 2 for sample sizes of 28 and 30.

## Discussion

The present data clearly affirm that the temporal muscle is responsible for a large artifact which can be induced by clenching the teeth and that this artifact has influence on the signal-to-noise ratio of fNIRS measurements. Experiment 1 shows that clenching the teeth results in high-amplitude increases in O_2_Hb and decreases in HHb with a time course of several tens of seconds over the temporal area. As expected (see Introduction), O_2_Hb and HHb trajectories of the muscle artifact mirror the typical trajectories of neural activity (Plichta et al., 2007b; Towse et al., 2011). This artifact is correlated with head size which fits to the assumption that larger head sizes are associated with larger muscles. Thus, head size might be considered in future fNIRS studies covering temporal areas. This is also of relevance, as frontal areas showed the reversed pattern with O_2_Hb decreases and HHb increases. We interpret this as redistribution of the hemoglobin from non-activated to activated tissue layers on the skull. As hemoglobin is reallocated from areas covering different layers (skin and the frontal muscle), the trajectory is not completely anti-correlated. Redistribution is a phenomenon which is known from cortical activation (Harel et al., 2002) which might also be true for skin and muscle blood flow. Depending on head size, reallocation effects might depend on the size of the temporal muscle. Decreases in frontal areas are insofar of relevance as different paradigms of frontal cortex activity reported decreases of O_2_Hb (Kopf et al., 2011). It can be speculated that the temporal muscle artifact may also be responsible for changes in frontal blood flow in cognitive tasks of the prefrontal cortex.

A further finding is that before the large anti-parallel changes in O_2_Hb and HHb an initial parallel dip of both chromophores over the temporal and frontal area could be observed. We have one technical, one physiological and one neuronal assumption but both of them cannot explain the data sufficiently. The parallel dip of O_2_Hb and HHb is probably induced by the movement of the jaw as subjects were instructed to clench the teeth for 2 s. Cui and colleagues argued that parallel signal trajectories of O_2_Hb and HHb are induced by noise due to head movement (Cui et al., 2010). In our opinion, movements of the head but also movements of the temporal muscle lead to movements of the optodes resulting in parallel signal trajectories of O_2_Hb and HHb. Clenching the teeth results in palpable movements of the skin over the temporal cortex. However, the initial dip can also be observed in the muscle BOLD signal which speaks against a technical artifact due to probe-set movement. Muscle contractions are associated with short drops in blood volume followed by a hemodynamic response (Towse et al., 2011). However, this explanation cannot explain the initial dip in frontal areas outside the temporal muscle. A third idea might be the activity of the default mode network which has one hub in the medial frontal areas. This may be best investigated in a group fMRI study not only in one single subject.

Based on the trajectory and localization, the temporal muscle artifact can be detected by visual inspection. We also conducted some further peripheral measurements probing the capacity of EMG and SOD to map the muscle artifact (Table 1). In sum, correlations of EMG and SOD activity with the fNIRS artifact did not provide convincing evidence that EMG and SOD signals as indicated by average, peak and latency of peak activity can predict the fNIRS artifact. Only EMG peak and O_2_Hb peak and SOD latency and O_2_Hb/HHb latency showed significant positive correlations. On a single subject level, 19 out of 31 subjects showed positive associations between SOD and fNIRS artifact with correlation coefficients above 0.5. These findings highlight the need for regression of peripheral measures on a single subject level rather than on a group level for efficient artifact correction. The correlation with head size as covariate did results in the same findings indicating no influence of head size. Future studies should optimize the localization or increase the used numbers of EMG electrodes and SOD optodes for more valid estimations of the temporal muscle artifact. This is particularly of relevance as it can be speculated that also smaller artifacts induced by more subtle temporal muscle movements might contaminate the fNIRS signal. Future work should systematically investigate the intensity of muscle activity on fNIRS signals in a parametric design using electric or magnetic stimulation of temporal muscle.

Even if artifacts with minor amplitude are considerable, the provoked large artifact of experiment 1 as induced by clenching the teeth can be visually identified. Visual inspection was supported by correlation analyses of fNIRS signal over the temporal muscle with gray matter and muscle BOLD signals in experiment 2. In a resting state fNIRS-fMRI measurement of one subject, we could identify two time windows contaminated with the temporal muscle artifact. For the signal outside these time windows, the fNIRS signal correlated to a greater extent with gray matter signal (gray-shaded epochs in Figure 2B). Within this time window, correlation was higher for the muscle BOLD signal (green-shaded epochs in Figure 2B). The fMRI-fNIRS study in experiment 2 demonstrated that it is possible to measure brain activity over the temporal muscle. However, during the artifact it seems difficult to measure brain activity as the fNIRS signal probably is completely saturated by the muscle artifact. This is clear evidence that the temporal muscle artifact has influence on the signal-to-noise ratio of fNIRS measurements.

In experiment 3, we analyzed an existent dataset (Schecklmann et al., 2014) of sound-induced activity in auditory cortex and Broca's area. Based on the information of experiment 1 with respect to the trajectory and the localization of the artifact, we detected temporal muscle artifacts and divided the subjects into two groups with and without artifacts. For the block design half of the subjects, in the event-related design 60% of the subjects showed artifacts indicating the temporal muscle artifact as a frequent event in standard fNIRS measurements. Noisy blocks/events in the subjects with artifacts were excluded in a separate step. Based on the anatomical ROIs, we could demonstrate sound-evoked activity in the auditory ROI for HHb for the event-related design, for O_2_Hb and HHb in auditory and Broca's ROI for the block-design using a simple HRV model. This model estimates transient increases of activation during the block design which was the case in the auditory ROI and the Broca ROI showing increases in activity within the first 10 s of sound stimulation which itself lasted 20 s. Transient increases might be explained by the perception of the sound as a continuous stimulus resulting in transient activation (Harms and Melcher, 2002; Gutschalk et al., 2010). An additional interesting finding is that O_2_Hb and HHb show an event-related parallel run in the inferior anterior channels indicating a capillary dominated generation of the hemodynamic response (for detailed discussion see Yamamoto and Kato, 2002; Ehlis et al., 2005). The trajectories in this area were stable over the whole stimulation period. Thus, we used model-based analyses using a boxcar function of 15 s.

Contrasting subjects with and without artifacts and contrasting conditions with and without exclusion of artifacts showed effects of artifacts for HHb in the right auditory ROI for all simple HRV models and for O_2_Hb in both hemispheres in the Broca ROI for the block design. Even if these findings are based on effect size calculations as contrasts were not significant, they highlight a putative role of the temporal muscle artifact in sound-evoked fNIRS activity. Inspecting the effects of excluding artifacts for the whole sample shows that the temporal muscle artifact might play a smaller role in large sample sizes. But even for large sample sizes *t*-values in Figure 5 indicate that artifacts affect fNIRS measurements in a way that non-significant (*t*-values < 2) contrasts get significant (*t*-values > 2) and vice versa. These findings again indicate that the temporal muscle artifact has influence on the signal-to-noise ratio of fNIRS measurements.

For future studies, temporal muscle artifacts should be prevented by instructing the subjects not to clench their teeth. Possible interventions to prevent clenching the teeth might be: (1) not to swallow as swallowing is often accompanied with clenching the teeth; (2) to swallow by avoiding clenching the teeth which can be demonstrated and trained in a short session before the measurement; (3) to open the mouth during the whole measurement; (4) or to use spacers between the incisors (Schecklmann et al., 2011). For the future, fronto-temporal fNIRS signals should also be visually inspected for temporal muscle artifacts and contaminated blocks/events should be excluded. Large sample sizes seem to prevent effects in signal-to-noise ratio by this artifact. Available artifact correction methods such as principal or independent component analyses (Virtanen et al., 2009) might be capable of correcting contaminated data. Usefulness of peripheral measures such as EMG or SOD channels for artifact correction needs to be further evaluated.

## Author contributions

MS, FH, AE, and AF contributed to the conception and design of the work. AM, and FH acquired the data. FH was responsible for data analyses. MS, FH, BL, AM, AE, and AF contributed to the interpretation of the data. All authors was involved in drafting or revising the work for important intellectual content, in the final approval of the version to be published. All authors agree to be accountable for all aspects of the work in ensuring that questions related to the accuracy or integrity of any part of the work are appropriately investigated and resolved.

### Conflict of interest statement

The authors declare that the research was conducted in the absence of any commercial or financial relationships that could be construed as a potential conflict of interest.
